# Exploratory mixed methods analysis of self-authored content from participants in a digital alcohol intervention trial

**DOI:** 10.1186/s13011-023-00569-4

**Published:** 2023-10-28

**Authors:** Elizabeth S. Collier, Jenny Blomqvist, Joel Crawford, Jim McCambridge, Marcus Bendtsen

**Affiliations:** 1 Department of Health, Medicine and Caring Sciences, Linköping University, Linköping, 581 83 Sweden; 2 Division of Bioeconomy and Health, Perception and Design Unit, RISE Research Institutes of Sweden, Stockholm, 114 28 Sweden; 3 Department of Health Sciences, University of York, York, United Kingdom

**Keywords:** Alcohol, Intervention design, Mixed methods, Behavioural change, Prompts

## Abstract

**Background:**

Digital interventions readily permit data capture of participant engagement with them. If future interventions are intended to be more interactive, tailored, or a useful resource offered to users, it may be valuable to examine such data. One module available in a digital alcohol intervention recently tested in a randomised control trial offered participants the opportunity to self-author prompts that were sent to them by a text message at a time of their choosing. This study thus aimed to evaluate these self-authored prompts to increase knowledge on how individuals negotiate behaviour change and assess whether intervention content can be improved in the future.

**Methods:**

The self-authored prompts were evaluated qualitatively using a combination of content and thematic analysis. The identified themes and subcategories are exemplified using anonymized quotes, and the frequency that each identified theme was coded for among the prompts was calculated. Associations between baseline characteristics and the odds of authoring a prompt at all, as well as a prompt within each theme, were investigated using logistic regression.

**Results:**

Five themes were identified (*Encouragement Style, Level of Awareness, Reminders of reasons to reduce/quit, Strategies to reduce/quit*, and *Timescale*), all with several subcategories. The prompts module was more likely to be used by women and older individuals, as well as those for whom reducing alcohol consumption was perceived as important, or who felt they had the know-how to do so. Participants who had immediate access to the support tool (intervention group) were more than twice as likely to author a prompt (OR = 2.36; probability of association > 99%) compared to those with 4-month delayed access (control group).

**Conclusions:**

Individuals who engaged with the prompts module showed evidence of using the information provided in the support tool in an active way, with several showing goal setting and making plans to change their drinking behaviour. Individuals also used this opportunity to remind themselves of personal and specific reasons they wanted to change their drinking, as well as to encourage themselves to do so.

**Supplementary Information:**

The online version contains supplementary material available at 10.1186/s13011-023-00569-4.

## Background

Regular consumption of alcohol is associated with an increased risk of negative physical and psychological consequences, as well as harm to others and society more broadly[1–3]. Behavioural change interventions are one option for supporting individuals to reduce their alcohol consumption [4]. In countries where internet connectivity and mobile phone ownership are widespread, interventions can be disseminated digitally, reducing pressure on primary healthcare to provide such support. Although the evidence supporting digital alcohol interventions in treatment of excess alcohol consumption is not yet conclusive [5, 6], reviews indicate that such interventions can lead to reductions in total weekly alcohol consumption and episodes of heavy drinking [7]. Offering alcohol interventions digitally could also confer additional benefits such as increased accessibility and reduced stigma [8]. Nonetheless, there is room for improvement regarding the effectiveness of digital interventions, which can be negatively impacted by factors such as risk of attrition [6]. Digital interventions differ in their design and content, as well as how this content is delivered, and features and modules vary in their perceived usefulness and satisfaction among users [9]. A common feature is the use of prompts—reminders or advice that are sent by email, text message, or push notification—which aim to support behaviour change through providing motivational messages to increase self-efficacy, and tips to increase knowledge of how to change.

A digital support tool was recently developed and tested in Sweden by Bendtsen and colleagues [5, 10] in a randomized control trial (RCT) [10]. There, 2129 individuals with harmful alcohol use (aged 18 + years and with access to a mobile phone) were randomized to either a control (N = 1066) or intervention (N = 1063) group. The results [10] indicated that the intervention group reported reduced alcohol consumption relative to the control group at both 2- and 4-month follow-up in terms of total weekly consumption (incidence rate ratio (IRR) = 0.89) and heavy episodic drinking (IRR = 0.77). The tested support tool [5, 10, 11] included a module where participants were invited to self-author prompts that they may find useful. This optional module (hereafter referred to as the prompts module) took the form of writing a text message to themselves, which was then delivered on a date and time of their choosing in the coming week (up to three times). This could for instance be a reminder of their desire to change or a plan for dealing with a particular situation that could be anticipated in advance. Although this was, to the best knowledge of the authors, a novel module, it was hypothesised that it would leverage behavioural change techniques [11] relating to problem solving and goal setting [12] for which there is evidence that their inclusion in interventions may support behavioural change. Specifically, participants were prompted to 1) write messages that reminded them of their commitment and 2) have these sent to themselves at times when they anticipated that there may be environmental triggers to drink. Thus, the intention behind the prompts module was to offer participants a resource in planning for coping with predictable behavioural triggers, encourage problem solving, and reinforce earlier intentions [5].

Here, we present an exploratory analysis of the self-authored prompts collected during the parent trial [10, 12], whose content may offer insights into how individuals’ express their motivations, readiness to change, self-reflection, and the various strategies they may employ to reduce their drinking. Such insight could indicate how well intervention content reflects how participants negotiate behavioural change with themselves and highlight whether the content and information provided is in alignment with what those trying to reduce their drinking focus on and reflect over. This may be relevant for intervention design because recent additional analysis of data from the same parent trial suggests that study participation can improve know-how and confidence to reduce alcohol consumption and that this mediates behavioural change [13]. Previous work investigating user-experiences among the intervention group in another RCT targeting alcohol consumption also indicated that self-reflection over their drinking appeared to be an important driver of reduced consumption [14]. As such, designing interventions that contribute to or facilitate a boost in confidence and know-how or induce self-reflection is of relevance. The primary objective of the present work was thus to qualitatively investigate the content of self-authored prompts to obtain insight into such self-reflection, and general negotiation of behavioural change as a process, as described by individuals themselves during a trial.

Further, if alcohol interventions are to become more individualized and tailored in the future, as has been proposed as a possible way to improve effectiveness [15], assessing for whom given intervention content, e.g., a prompts module, is appealing to would be fruitful. A secondary objective was thus to test whether baseline characteristics were associated with the likelihood of participants engaging with the prompts module at all, or were related to the types of prompts that were written.

As a waiting-list design was employed in the parent trial, the possible implications of this for engagement with the prompts module as well as the types of prompts authored were also explored (i.e., contrasts between the intervention group who were given immediate access to the support tool and the control group who waited four months before receiving access to it were explored). This was deemed of interest as the implications of waiting-list designs are not yet fully understood [12, 16] although it has been suggested that individuals with increased readiness to change may be particularly negatively impacted by being put on a waiting list for support tool access [17]. Variations in how the intervention and control group engaged with the prompts module may elucidate ways in which a waiting list design affects how those made to wait may engage with support tools they are subsequently given access to, beyond assessing drinking behaviour alone.

## Method

### Trial design

The parent study was a two-arm, double-blind, parallel group randomised effectiveness trial of a digital alcohol intervention. The trial was prospectively registered (ISRCTN48317451) and a trial protocol including a statistical analysis plan was made available prior to trial commencement [5]. Full methodological and intervention design details are provided in the Appendix.

### Participants

The target population of the main trial was Swedish adults seeking help online to reduce their alcohol consumption. Individuals were required to be at least 18 years of age, have access to a mobile phone, and be classified as having harmful alcohol use according to Swedish guidelines. At the time of data collection, this was defined as either drinking more than 9 (female) or 14 (male) standard drinks of alcohol per week (total weekly consumption) or drinking more than 4 (female) or 5 (male) standard drinks on a single occasion at least once a month (heavy episodic drinking). A standard drink is in Sweden defined as 12 g of alcohol. All study materials were in Swedish, and so individuals who did not comprehend Swedish well enough to understand these were implicitly excluded.

Participants were recruited to the trial using web search engine advertisements (Google, Yahoo, Bing) and Facebook. Individuals interested in the study sent a text message to a dedicated phone number. Within 10 min, they received a text message with a hyperlink that took them to a Web page asking for informed consent. Those who consented were asked to respond to a baseline questionnaire (which also assessed eligibility). The questionnaire included questions on demographics, current alcohol consumption, and three single item measures of confidence in one’s ability to reduce drinking (confidence), perceived importance of reducing drinking (importance), and knowledge of how to reduce drinking (know-how). Eligible participants were randomised after responding to the baseline questionnaire to either receive the novel digital alcohol intervention or to a control group. Drinking behaviour (weekly consumption and episodes of heavy drinking) was measured at 2-month and 4-month follow-up. The control group were provided with information regarding the risks associated with alcohol consumption and informed that they would receive access to the support tool after four months (waiting list design). There were no other differences between the control and intervention groups apart from the control group being given access to the novel intervention following completion of the 4-month follow-up. The demographics of the full cohort and those who engaged with the prompts module (N = 476 respondents) within each group (intervention and control) are shown in Table 1.

**Table 1:** Baseline characteristics of total participants in the intervention and control groups, and those who used the prompts module

	Intervention (cohort) n = 1063	Intervention (prompts) n = 318	Control (cohort) n = 1066	Control (prompts) n = 158
Total weekly consumption past week, median (IQR)	16 (10;25)	16 (10.25;25)	17 (10;25)	17 (10;25)
Episodes of heavy drinking past month, median (IQR)	6 (4;12)	7 (4;12)	6 (4;10)	6 (4;12)
Age, median (IQR)	46 (36;54)	48.5 (42;57)	45(35;55)	49 (43;55)
Sex, n (%)				
Female	612 (58%)	212 (67%)	625 (59%)	111 (70%)
Male	451 (42%)	106 (33%)	441 (41%)	47 (30%)
Household characteristics, n (%)				
Not living alone with kids	282 (36%)	111 (35%)	373 (35%)	64 (41%)
Not living alone no kids	267 (25%)	83 (26%)	277 (26%)	40 (25%)
Living alone with no kids	219 (21%)	60 (19%)	224 (21%)	26 (16%)
Living alone with kids	114 (11%)	41 (13%)	101 (9%)	15 (9%)
Partner but not living together	80 (8%)	23 (7%)	91 (8%)	13 (8%)
Importance, median (IQR)	10 (9;10)	10 (10;10)	10 (9;10)	10 (10;10)
Know-how, median (IQR)	5 (2;6)	5 (3;7)	5 (2;7)	5 (3;7)
Confidence, median (IQR)	6 (5;8)	6 (5;8)	6 (5;8)	6 (4.25;8)

### Outcomes

One component of the intervention was to offer participants the opportunity to author their own prompts that they would receive at a time and date of their own choosing (up to three times a week). This could for instance be a reminder of their desire to change their behaviour or a plan for when they are confronted with a behavioural trigger. Authoring prompts was an optional component of the intervention. For the present work, the self-authored prompts themselves are a primary outcome, in the form of written qualitative data. Whether or not participants authored any prompts while having access to the intervention tool was a secondary outcome. In total, 1166 prompts were authored.

### Data analysis

It should be acknowledged that all analyses herein were exploratory and were not described in the trial protocol. This was due to the researchers not anticipating that the prompts module would be so frequently used as it turned out to be.

### Qualitative analysis

The qualitative approach taken is best described as a combination of content and thematic analysis [18]. Aspects of content analysis that were relevant include describing the characteristics of the prompts based on the actual words used and the frequency that similar terms appear, as well as the identification of recurring keywords. The positioning of content analysis as appropriate for exploring relatively unknown phenomena [18] meant that this approach was more relevant in the earlier steps of the analysis. Aspects of thematic analysis that were employed were assessing how the codes grouped into broader concepts (based both on manifest content and behavioural theory), and extrapolation of underlying concepts that were not necessarily explicitly stated. This approach was more relevant in the later steps of the analysis. The qualitative analysis was conducted on text-only data in the original written language and coders were blinded to randomization status.

The prompts were read and re-read by a single researcher (ESC), who generated notes and an initial code list and codebook. The initial codebook was reviewed by a second researcher who was familiar with the dataset (MB), for clarity and comprehensiveness. The data were then coded in RQDA (R package for Qualitative Data Analysis [19]) according to the initial codebook. There was no limit on the number of codes for a single prompt. During this stage, annotations and notes pertaining to ways that the codes could potentially be grouped based on both semantic meaning and patterns of applying the codes (e.g., recurring instances of codes clustering together) were made. Some codes were merged, some were discarded, and new codes were added. The codebook was updated accordingly and reviewed alongside the fully coded dataset by MB.

After a joint discussion on the codebook and code applications, where further adjustments and clarifications were made, the codes were conceptually grouped which gave rise to an initial list of candidate themes. Codes that did not seem to fit within these groupings were retained during the next round of coding. During this step, the coding process was more carefully guided by attempting to identify broader concepts associated with capability, opportunity, and motivation, as well as intentions and evidence of behavioural planning and application of strategies for alcohol reduction. The codebook was again refined, including renaming, merging, and removing codes (particularly those which did not appear to fit with any of the wider identified concepts due to being highly specific, or not substantially present throughout the dataset). Prompts which remained uncoded at this stage were not considered further in the analysis (N = 88).

The codebook was then used by a third researcher (JB) to independently code the full dataset. Following this, based on discrepancies in coding between coders, further codebook refinements were made and the dataset was coded an additional time jointly ESC and JB. The final coded dataset was assessed and agreed upon by the researchers. The codebook is provided in the Appendix, Table A1.

### Quantitative analysis

To transform the qualitative data into a form suitable for quantitative processing, the presence (1) or absence (0) of each code for every prompt was recorded. Thereafter, the presence or absence of each thematic category was noted for each participant depending on the subcategories assigned to all the prompts they had authored.

Logistic regression was used to assess if any baseline characteristics (age, gender, household characteristics, weekly alcohol consumption, episodes of heavy drinking, confidence, importance, know-how) as well as group allocation (intervention vs. control) were associated with an increased odds of engaging with the prompts module. Logistic regression was also used to estimate the odds ratio of authoring a prompt belonging to a specific thematic category given baseline characteristics or group allocation. Models were estimated using the *quap* function from the *rethinking* package [20] in R (version 4.1.1). Standard normal priors (µ = 0; σ = 1) were used for all coefficients.

## Results

**Fig. 1:**
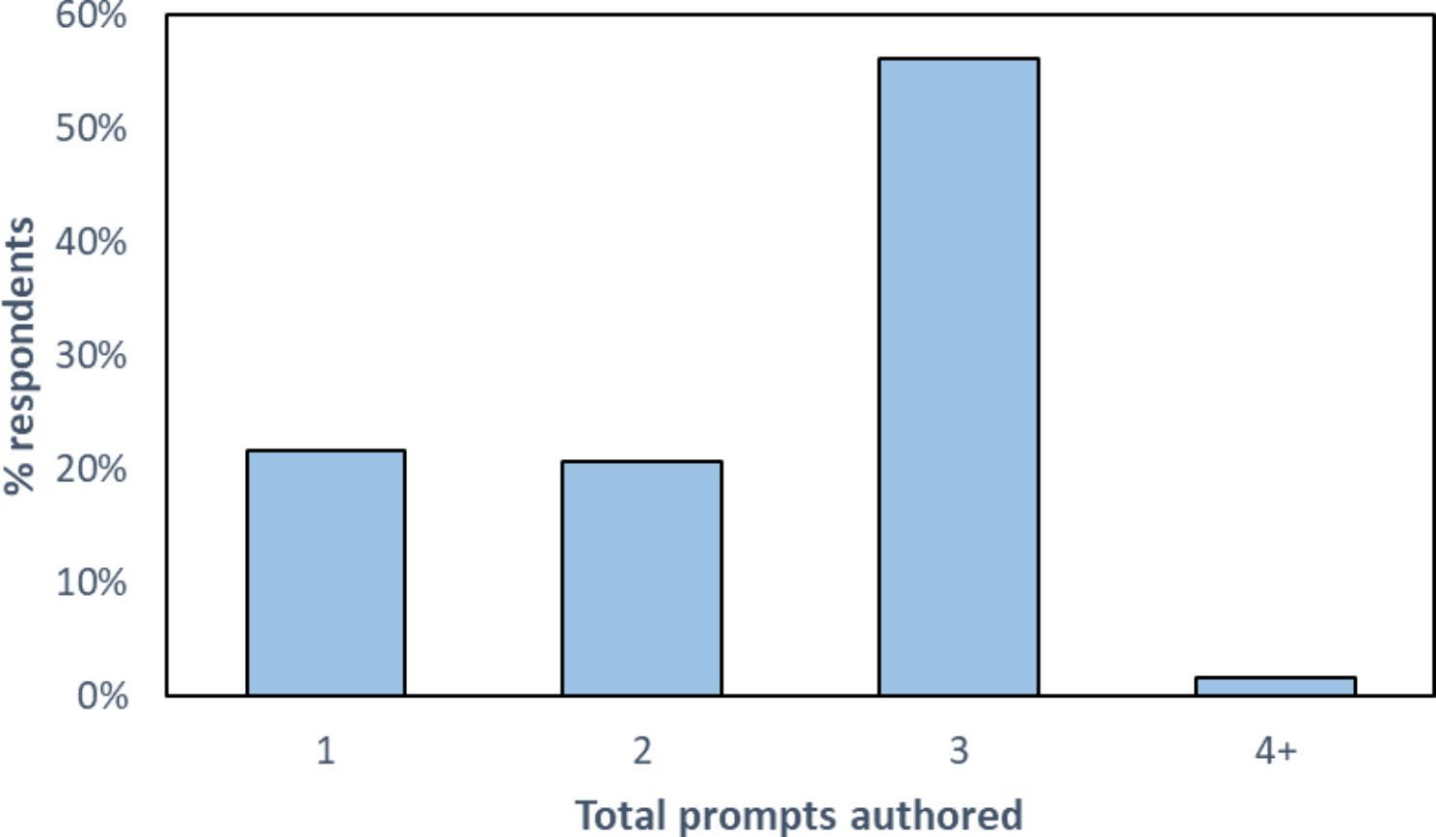
Shows the percentage of respondents who authored one, two, three, and four or more prompts while having access to the support tool

As shown in Fig. 1, most respondents wrote 1–3 prompts, with 3 being the most common, while very few authored four or more prompts while having access to the support tool.

### Thematic content of self-authored prompts

**Fig. 2:**
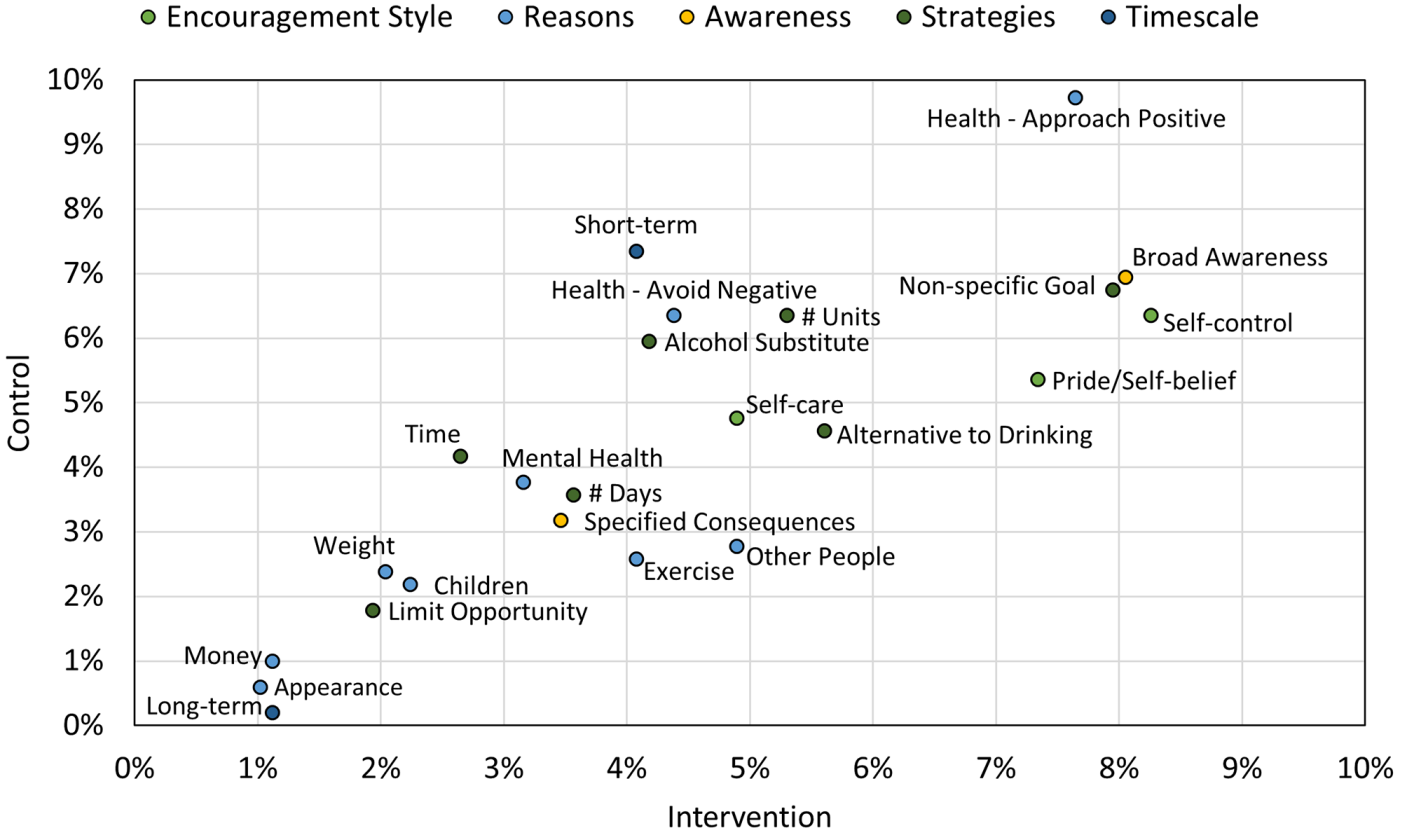
Diagram showing the frequency of codes appearing in the intervention and control groups

As shown in Table 2, the most frequently coded for subcategory was *Health - general intention/desire to improve physical or emotional well-being*, whilst the least frequently coded for subcategories were *money* and longer-term thinking. Five thematic categories (themes) were identified the dataset, each with subcategories, see Table 2. The frequency that each subcategory appeared in both the control and intervention group is shown in Fig. 2.

**Table 2:** Thematic categories and subcategories detected through qualitative analysis and the total frequency (%) of prompts in each. *Note*: Percentages are rounded and do not sum to exactly 100%.

Thematic category	Subcategories
Encouragement style	*Pride/ self-belief (129, 7%)*
	*Self-care (89, 5%)*
	*Self-control (145, 8%)*
Level of awareness (Awareness)	*Specified consequences (67, 4%)*
	*Broad awareness (147, 8%)*
Reminders or reasons to reduce/quit (Reasons)	*Appearance (17, 1%)*
	*Exercise (73, 4%)*
	*Health – general avoidance of negative physical/emotional wellbeing (86, 4%)*
	*Health – general intention/desire to improve physical or emotional wellbeing (160, 8%)*
	*Health - secondary prevention (19, 1%)*
	*Weight (44, 2%)*
	*Mental health (61, 3%)*
	*Money (19, 1%)*
	*Other people (73, 4%)*
	*Other people – children (38, 2%)*
Strategy to reduce/quit (Strategy/goal)	*Non-specific goal (153, 8%)*
	*Limit opportunity (32, 2%)*
	*Moderation strategy – days (74, 4%)*
	*Moderation strategy – units (129, 7%)*
	*Moderation strategy – time (54, 3%)*
	*Alternative to drinking (108, 6%)*
	*Substitution with alcohol replacement (89, 5%)*
Timescale	*Longer-term thinking (16, 1%)*
	*Shorter-term thinking (106, 6%)*

In the sections that follow, the thematic categories are described and explored, with anonymized quotes provided to exemplify these and their associated subcategories.

#### Thematic category 1: encouragement styles

The category *Encouragement Style* encompasses instances where respondents were imparting some degree of supportive message or call to action to themselves. Three subcategories were identified: *Self-Control* (urging themselves to retain or take control over their alcohol consumption), *Self-Care* (urging themselves to put themselves first, to be kind and gentle on themselves, or take care of their own physical/mental state), and *Pride/Self-Belief* (displaying that they are or should be proud of themselves, or that they believe in their ability to reduce their drinking). Overall, *Self-Control* was more prevalent than the other two encouragement styles, and both *Self-Control* and *Pride/Self-Belief* were more common among the intervention group than the control group. The three *Encouragement Styles* are exemplified below in descending order of frequency (*Self-Control, Pride/Self-Belief, Self-Care*). The *Encouragement Styles* were sometimes co-coded, in particular *Self-Control* and *Pride/Self-belief.*

*“Avoid [alcohol] today, Thursday!!! It is NOT worth the tiredness during Friday’s workday. Keep going!”*

*“Fight for my goal to lose weight, dare to change my patterns. I can and I will”*

*“You feel better today right? Nice to avoid anxiety and being drunk from spirits, right? Take care of yourself!”*

#### Thematic category 2: level of awareness

Many participants wrote prompts conveying some *Level of Awareness* regarding the impact their drinking has had or is having on their life or others around them. Two subcategories were identified: *Broad Awareness* and *Specified Consequences.* The key difference between these is that *Broad Awareness* indicates a general sense of negativity towards their current behaviour or awareness that there can be consequences to drinking excessively, whilst *Specific Consequences* involves specifying some direct impact of drinking on other decisions and behaviours (e.g., sending texts that are regretted later), goals (e.g., getting fitter or losing weight), and people (e.g. negatively impacting a relationship), or stating some direct consequence of drinking (e.g., poorer performance at work). *Broad Awareness* was coded for more often than *Specified Consequences* overall. The representation of *Broad Awareness* was higher among the intervention than control group, whilst *Specified Consequences* appeared at a similar frequency in both groups. Although prompts coded as *Broad Awareness* ranged from short reflections on their behaviour to lengthier consideration of the reasons they drink or implications that they are dissatisfied with their current circumstances, they lacked specificity. *Specified Consequences* could in some ways be considered the next step succeeding *Broad Awareness.* Nonetheless, it seems reasonable that—akin to individuals typically moving along the stages-of-change in a non-linear pattern—there would be some iterative movement between the two, and it should be emphasized that the self-authored prompts here provide only snapshots of respondents’ reasoning. Examples of *Broad Awareness* and *Specified Consequences*, respectively, are:

*“It’s ok! You don’t need alcohol to reward yourself or to drink away the difficult things!”*

*“Remember not to drink too much. It leads to bad decisions and damages relationships”*

Prompts coded as *Broad Awareness* were not coded alongside codes within the *Strategies* category often but this did occur, typically when the prompt consisted of multiple sentences. For example, the following prompts were coded as *Broad Awareness* alongside *Limit Opportunity* and *Strategy – Units* respectively.

*“Don’t go to the off-licence! You’ll see what a difference there is after a week without alcohol.”*

*“Buy max 4 beers at the off-licence this afternoon! It’s time to take things in hand now!”*

Like *Broad Awareness, Specified Consequences* also sometimes appeared alongside codes from *Strategies.* However, it was more common that *Reasons* were co-coded here. This is logical: specifying the consequences of drinking requires overtly recognizing other behaviours or individuals that are impacted by the person’s drinking. The most common *Reasons* coded alongside *Specified Consequences* were *Health* (both avoid negative consequences and approach positive outcomes), *Mental Health*, and *Other People*, exemplified respectively here:

*“Get rid of thoughts about reward or stress. It only leads to anxiety and bad text messages.”*

*“Alcohol gives you unbearable anxiety. It’s not worth it, you know that.”*

*“You will stop drinking for your own well-being and for your kids’ sake. They don’t like seeing you unsober.”*

#### Thematic category 3: reasons

Respondents often used the prompts module to remind themselves of specific *Reasons* they wanted to change their drinking behaviour. Such prompts were often short, although in some cases respondents provided a list of several reasons. The most common reasons provided were related to health, with those conveying a desire to move towards improved health (*Health – Approach Positive*) being more prevalent than those mentioning avoidance of negative health consequences associated with alcohol consumption (*Health – Avoid Negative*). Reasons relating to money and physical appearance were the least common. The *Health – Approach Positive* subcategory includes mentions of desiring both better physical health and general well-being, e.g., when respondents referenced wanting to “feel better” more generally. Similarly, the *Health – Avoid Negative* subcategory includes prompts where respondents indicated wanting to avoid poor health outcomes and negative emotions. Allusions to general wellbeing were therefore not coded as *Mental Health* related reasons unless specific mental health concerns were mentioned (e.g., anxiety, depression) or the term “mental health” was explicitly used by respondents. Examples of *Health – Approach Positive* and *Health – Avoid Negative* are:

*“I want to feel better, have more energy, and get better health.”*

*“Don’t drink! Remember what happens and how damn bad you feel. Find something else! Go and train!”*

#### Thematic category 4: strategy/goal

Several prompts suggested that respondents were already or were planning to employ some form of *Strategy/goal* to moderate their drinking. This took many forms, including *Limit Opportunity* for drinking (avoiding the pub, not keeping alcohol at home), moderating consumption by limiting or counting *Days, Units*, and/or *Time*, suggesting an *Alternative to Drinking* (such as taking a walk or some other activity), and *Substitutions with Alcohol Replacement* (including non-alcoholic versions of drinks, and water), as well as *Non-Specific Goals* where no specific limitations were provided but respondents seemed nonetheless to have some goal in mind. Among these, attempting to limit the number of *Units* consumed or setting a *Non-specific Goal* were the most commonly observed, whilst *Limit Opportunity* was the least common. Suggesting an *Alternative to Drinking* and mentioning a *Non-Specific Goal* were more common among the intervention group whilst setting goals pertaining to *Units* and *Time* and using *Substitutes* for alcohol, were more common among the control group. It should be noted that stating a *Non-specific Goal* does not necessarily mean that these individuals did not have a concrete goal, only that they did not specify it in their prompts. Examples of *Non-Specific Goal*, moderation through limiting *Units*, and suggesting an *Alternative to Drinking* are:

*“Now you have to think about how many glasses you will have today.”*

*“Remember! Max two glasses per day, to feel better and avoid negative effects.”*

*“Take a long walk, then a cup of tea and knitting! Tomorrow is a new day! Feel well!!”*

#### Thematic category 5: Timeline

Regarding the *Timeline* category, there was a clear preference among both groups to focus on the short-term rather than the long-term, and prompts coded as *Short-term Thinking* were also observed more often among the control group. Many prompts coded as *Short-term Thinking* involved participants reminding themselves to just not drink that day and were typically quite short. Although not many prompts were coded as *Long-term Thinking*, most of those that were came from the intervention group, possibly reflecting a more exploratory, less focused approach to change upon being given immediate access to the tool.

*“Uh huh, so it’s Friday again. Think how lovely it would be to wake up on Saturday without a hangover.”*

*“Think about the rest of your life.”*

### Associations between baseline characteristics and self-authored content

The odds ratios of whether a participant authored any prompt, as well as whether they authored prompts belonging to each thematic category, are summarized in Table 3. The analysis suggests that those in the intervention group were more likely to use the prompts module (29.9% of intervention group participants) than those in the control group (14.8% of control group participants). Prompts coded as *Strategies, Reasons, Timescale*, and, to a lesser degree, *Level of Awareness* were more likely to be authored by the control group than the intervention group, whilst the intervention group seemed slightly more likely to author prompts coded as an *Encouragement Style.* Women and older individuals appeared more likely to engage with the prompts module as well as individuals with higher baseline importance and know-how scores. Living with children (with or without a partner) also showed an increased odds of writing a prompt relative to living alone without children. Baseline drinking metrics did not substantially affect the odds of authoring a prompt, although higher episodes of heavy drinking did seem related to somewhat higher odds of authoring prompts categorized as *Awareness* or *Reasons* as well as decreased odds for prompts categorized as a *Strategy.*

**Table 3:** Medians of the posterior distributions of odds ratio (with 2.5% and 97.% percentiles) indicating associations with the odds of authoring any prompt, and prompts that fall into each thematic group, alongside the posterior probability of association

	Median of posterior distribution (2.5%; 97.% percentiles) *Probability of association (OR < or > 1 in the direction of the median)*					
	Any prompt	Encouragement style	Level of awareness	Reasons	Strategy/goal	Timescale
Group (intervention vs. control)	2.36 (1.92; 2.93) *> 0.99*	1.06 (0.83; 1.37) *0.68*	0.87 (0.64; 1.19) *0.81*	0.86 (0.71; 1.03) *0.95*	0.80 (0.66; 0.96) *0.99*	0.52 (0.36; 0.75) *> 0.99*
Total weekly consumption	1.00 (0.99; 1.01) *0.40*	1.00 (0.98; 1.01) *0.41*	1.00 (0.98; 1.02) *0.34*	1.00 (0.99; 1.01) *0.44*	1.00 (0.99; 1.02) *0.85*	0.99 (0.96; 1.02) *0.81*
Episodes of heavy drinking	1.00 (0.98; 1.02) *0.43*	1.00 (0.98; 1.02) *0.38*	1.02 (1.00; 1.05) *0.96*	1.01 (1.00; 1.03) *0.91*	0.98 (0.97; 1.00) *0.98*	1.02 (0.98; 1.05) *0.85*
Sex (man vs. woman)	0.57 (0.45; 0.71) *> 0.99*	0.69 (0.52; 0.91) *> 0.99*	0.70 (0.49; 1.00) *0.97*	0.65 (0.52; 0.80) *> 0.99*	0.85 (0.69; 1.06) *0.93*	0.63 (0.40; 0.98) *0.98*
Age	1.03 (1.02; 1.04) *> 0.99*	0.98 (0.97; 0.99) *> 0.99*	0.97 (0.95; 0.98) *> 0.99*	0.98 (0.97; 0.99) *> 0.99*	1.00 (0.99; 1.01) *0.53*	1.00 (0.98; 1.02) *0.46*
Alone with kids vs. alone no kids	1.32 (0.98; 1.77) *0.96*	0.99 (0.62; 1.61) *0.51*	1.06 (0.59; 1.87) *0.58*	1.46 (1.04; 2.06) *0.98*	1.02 (0.71; 1.47) *0.54*	1.11 (0.51;2.39) *0.60*
Partner not living together vs. alone no kids	1.21 (0.76; 1.90) *0.80*	1.48 (0.88; 2.50) *0.92*	0.58 (0.28; 1.21) *0.08*	1.47 (1.00; 2.19) *0.97*	0.87 (0.56; 1.36) *0.73*	0.96 (0.39; 2.31) *0.47*
Partner living together no kids vs. alone no kids	0.99 (0.71; 1.36) *0.54*	0.91 (0.60; 1.39) *0.67*	0.87 (0.52; 1.46) *0.30*	0.93 (0.67; 1.30) *0.66*	1.07 (0.79; 1.45) *0.66*	1.00 (0.52;1.93) *0.50*
Partner living together with kids vs. alone no kids	1.34 (0.90; 1.97) *0.93*	0.95 (0.65; 1.38) *0.61*	0.71 (0.43; 1.14) *0.08*	1.35 (1.01; 1.80) *0.98*	1.02 (0.77; 1.35) *0.56*	0.98 (0.52; 1.79) *0.47*
Importance	1.09 (1.01; 1.18) *0.98*	1.17 (1.03; 1.33) *> 0.99*	1.09 (0.93; 1.29) *0.88*	1.10 (0.01; 1.21) *0.99*	1.02 (0.94; 1.10) *0.65*	1.07 (0.88; 1.30) *0.75*
Know-how	1.03 (0.99; 1.07) *0.95*	0.97 (0.92; 1.02) *0.91*	1.01 (0.96; 1.07) *0.57*	1.03 (0.99; 1.06) *0.92*	0.97 (0.94; 1.01) *0.94*	0.98 (0.90; 1.06) *0.73*
Confidence	0.98 (0.94; 1.02) *0.84*	1.00 (0.95; 1.06) *0.53*	1.04 (0.97; 1.11) *0.85*	1.01 (0.98; 1.06) *0.80*	1.01 (0.97; 1.05) *0.69*	1.03 (0.95; 1.13) *0.76*

Notes: For the variable “group”, odds ratios greater than 1 indicate an increased likelihood of authoring a prompt among the intervention group compared to the control group. For the sex variable, odds ratios greater than 1 indicate that being a man increases the likelihood of authoring a prompt. For the household status variable, odds ratios greater than 1 for each status indicate an increased likelihood of authoring a prompt relative to living alone with no kids. All other relationships show condition associations with the odds of authoring a prompt, where odds ratios greater than 1 indicate an increased odds given a one-unit increase above the mean in the variable tested assuming all else is equal. Analysis on the presence of any prompt was based on the full parent study cohort (N = 2129) while analysis on the presence of the five thematic categories was based on respondents to the prompts module (N = 476).

## Discussion

Insights on how individuals trying to reduce their alcohol consumption negotiate this behavioural change can be readily collected during digital intervention trials through, for example, modules inviting individuals to write prompts for their future selves. Such insight can be useful for improving intervention design by highlighting (mis)alignment in priorities (e.g., health vs. financial motivations), differences in favoured goal direction (e.g., approach positive vs. avoid negative consequences) and even how these vary by demographic factors. Such a prompts module was included in a digital support tool, the effectiveness of which was recently tested in a recent RCT conducted in Sweden [5, 10]. Through content and thematic analysis of the 1166 prompts that were self-authored by trial participants, we attempted to gain novel insights into these individuals’ engagement with their own behavioural change process.

Quantitative analysis of the factors associated with the odds of writing a prompt at all, as well as a prompt belonging to one of the five identified themes, provided data on who authored prompts as well as the frequency of different types of content. The patterns observed and how the results can contribute to future intervention design are discussed below. Because the data and approach are novel, there are limited comparisons possible with existing literature. The importance of reinforcing motivation to continue to pursue the behaviour change involved in reducing drinking, as well as the potential important role of reflection upon one’s own behaviour [14] have, however, been observed in previous studies [21].

Although engagement with the prompts module was not especially high, women and individuals living with children (with or without a partner), and older individuals were more likely to use this module. Interventions targeting these populations may therefore benefit from offering individuals the possibility to write their own prompts or keep a record of their experiences. Those who rated themselves as having higher know-how on reducing their alcohol consumption were also more likely to write a prompt, suggesting that this module may also be more attractive to those who already know what they may want to write, or what could help them change their behaviour. By extension, individuals with lower baseline know-how may be more reliant on the advice and strategies suggested within the support tool. Higher baseline know-how individuals were also more likely to author a prompt giving reasons to change but less likely to write one indicating the use of a strategy or setting a goal. While this could be interpreted counterintuitively, it seems equally possible that these individuals did not feel the need to specify their strategy for drinking less and were instead using the prompts module to remind themselves why they want to change. Individuals for whom reducing their drinking was important were also more likely to write a prompt relating to the reasons they want to change their behaviour, perhaps suggesting that this module had the added benefit of allowing them to remind themselves of the personal reasons why they perceive change as important.

It was observed that, as well being more likely to write a prompt at all, the intervention group were more likely to author prompts conveying pride/self-belief and self-control than the control group, whilst individuals in the control group were more likely to write a prompt suggesting goal setting or implementing a specific strategy to drink less. More diffuse subcategories (e.g., *Broad Awareness* and *Non-specific goal*) were also more common among the intervention than control group. These results could imply that there may be differences between groups both in terms of their status regarding behavioural change and how they experienced the support tool. Such differences are *prima facie* surprising, as the only difference across groups lies in being made to wait as a result of the study design (waiting-list design). By definition, all participants in the main RCT would fall somewhere between the contemplation and action stages of change, which are characterized by higher ambivalence (mental discomfort resulting from holding both positive and negative feelings towards some behaviour) relative to the pre-contemplation and maintenance stages [22]. However, the intervention group were immediately offered a tool designed to help guide them to resolving their ambivalence by reducing or eschewing drinking, whilst the control group were not. Since heightened ambivalence can increase people’s level of information processing [22] and attention paid to information relevant to resolving their discomfort [23], this may have implications for how the support tool was experienced and used by the control group upon gaining access, as well as their behaviour during the 4-month waiting period.

The experiences of the control group from the parent trial with which this work is associated are reported in detail elsewhere [16], and revealed that several (42%) reported feeling frustrated, irritated, or disappointed to be on the waiting-list (due to feeling ready to start changing). Nonetheless, 55% of respondents also reported deciding to motivate themselves to reduce their drinking despite being on a waiting list, and an additional 16% reported finding other support to help reduce their drinking [16]. This suggests that, during the waiting time, at least some individuals in the control group investigated strategies for reducing their drinking, possibly motivated by the need to resolve their ambivalence. These strategies may have later been revealed in the prompts they authored, especially given the additional time this group had to find strategies that were helpful for them. On the other hand, others in the control group may have resolved their ambivalence by drinking. Indeed, around 17% of control group participants reported deciding to continue drinking as usual until they gained access to the support tool, and 11% said that they gave up on reducing their drinking [16], lending support to this possibility. However, without measuring ambivalence, this explanation remains speculative.

Nonetheless, this issue highlights the relevance of understanding how individuals are affected by the information provided to them in a digital intervention setting. Specifically, the importance of providing information that helps people resolve their ambivalence in favour of moderating/avoiding alcohol consumption instead of inadvertently facilitating rationalising its consumption (or pushing individuals to behave defensively or feel discouraged) is emphasized. This is pertinent because, as noted above, individuals with lower know-how in changing their drinking habits may be more reliant on the information and advice provided in an intervention setting. For both groups here, approaching positive health goals was the most common *Reason* coded for – more so even than avoiding negative health outcomes due to drinking. This would seem to suggest that interventions using prompts may wish to find a good balance between positive and negative health messaging, instead of focusing too heavily on avoiding negative health outcomes. This may be especially pertinent as health messages that highlight the negative health outcomes can be minimised or ignored altogether [24]. This may be due to ambivalence towards the negative outcomes, which can be rationalised as an expected part of a drinking episode or the ‘price of a good time’ [25–27].

It is interesting that *Limit Opportunity* was the least common strategy detected. This illustrates a preference for moderation strategies that require exerting agency and self-control (e.g., moderation through goal setting) over changing the environment (e.g., not keeping alcohol at home or avoiding social situations where drinking would occur). Since an intended purpose of the prompts module was to encourage individuals to exercise agency, this may not be surprising. Nonetheless, participants in alcohol reduction trials can react poorly to the idea that they drink excessively [28], a notion which suggesting changes in the environment to avoid alcohol completely may reinforce by implying that the individual has little control over their drinking behaviour. As such, moderation strategies where individuals feel that they can still drink, but not to excess, may induce less mental discomfort and remain more appealing than those which remove the option entirely.

Although baseline drinking metrics did not seem to markedly affect the odds of authoring a prompt, more frequent episodes of heavy drinking were somewhat related to greater odds of authoring a prompt categorized under *Awareness* or *Reasons*, and lower odds of writing a prompt categorized as a *Strategy.* This may indicate that individuals with different patterns of drinking may need different types of support and have different aspects to their motivations for change. For example, advice relating to reminders of the acute negative consequences of alcohol consumption may be more useful for an individual seeking help with heavy episodic drinking behaviour but who otherwise does not drink regularly. Whereas, for an individual who consumes alcohol regularly and in a more consistent pattern, providing specific reduction strategies and daily/weekly limits may be more appealing. Tailoring the content and frequency of prompts according to baseline drinking patterns may improve intervention effectiveness [29], and this study suggests that allowing individuals to author their own prompts could offer a more potent means to achieving this. This could also contribute to the shift towards attempting to offer increased individualization in intervention design, thought to be appealing to users as well as potentially improve efficacy [15, 29], in line with the wider literature on adaptive designs [30].

### Limitations

That a large proportion (77.6%) of participants did not author prompts highlights that we cannot be sure that the dataset we obtained is representative of all participants from the parent trial. The risk of selection bias is an important study limitation because, in addition to the observed differences, those who chose to use the prompts module may be different from those who did not in unmeasured ways. It should also be noted that individuals in the control group represented here are likely to be those who are highly motivated to change their drinking behaviour, since they engaged with the support tool even after being on a waiting list for 4 months.

Some of the prompts were single words and somewhat difficult to code due to lack of context: many of the 88 prompts that remained uncoded were single words. To minimise over-interpreting the data, such short prompts were rarely assigned codes such as *Specified Consequences* and instead were instead typically coded as *Reasons* (e.g., “marriage”) and *Strategies* (e.g., “1 glass”). Even prompts that were not single words could often be very short with little to no context, thus limiting interpretation. For example, “Child-free weekend ahead! Good work this week” could in principle be coded with *Other People – Children* given that children are mentioned. This code was ultimately not applied in this case, however, since children did not seem to be a *Reason* to change. This prompt was instead coded with *Pride/Self-belief* (due to the self-congratulation in the second sentence) and no assumption was made about the section pertaining to children. Taken together, these aspects highlight that – despite the careful coding procedures used – the challenges inherent in working with this kind of material should be noted as a study limitation.

Finally, relationships between prompt behaviour (being a prompt engager or not, the type of prompts written etc.) and changes in drinking behaviour were not examined here because, although this would be an interesting line of research, the exploratory nature of the present work is not the ideal platform for such an endeavour. This is because the direction of any relationships detected would be ambiguous (does writing a prompt affect alcohol behaviour, or does a change in alcohol behaviour affect the type of prompt written), and any number of unmeasured variables could lead to erroneous inference. Instead, an experimental approach where individuals are randomized to produce prompts of varying themes/types would be more appropriate to investigate this, so that other factors could be adequately controlled for (e.g., demographics, baseline drinking behaviour etc.)

## Conclusions

Offering a module in a digital alcohol intervention where individuals could author their own prompts was appealing for some individuals, in particular women, older individuals, and those for whom reducing drinking was important and who felt they had the knowledge for how to do so. Qualitative evaluation of the content of these self-authored prompts suggests that the majority of respondents were engaging with the information provided via the support tool in an active way, with evidence of goal setting and making plans to change their drinking behaviour. Individuals also used this opportunity to remind themselves of specific and personal reasons they wanted to change their drinking, as well as to encourage themselves to do so.

### Electronic supplementary material

Below is the link to the electronic supplementary material.

Supplementary Material 1

## Data Availability

De-identified datasets generated during and/or analysed during the current study will be made available upon reasonable request to the corresponding author, after approval of a proposal and with a signed data access agreement.

## List of abbreviations

RCT: Randomised Control Trial
IRR: Incidence Rate Ratio
