# Supplementary material for: Exploratory mixed methods analysis of self-authored content from participants in a digital alcohol intervention trial
Source: Subst Abuse Treat Prev Policy. 2023 Oct 28;18:60. doi: 10.1186/s13011-023-00569-4 (PMC10613385; doi:10.1186/s13011-023-00569-4)
Supplement: Supplementary file 1 — Supplementary Material 1 [file 13011_2023_569_MOESM1_ESM.pdf]

APPENDIX

Exploratory mixed methods analysis of self-authored content from participants in a digital alcohol intervention trial

Elizabeth S. Collier[1,2*], Jenny Blomqvist[1], Joel Crawford[1], Jim McCambridge[3], Marcus Bendtsen[1]

[1] Department of Health, Medicine and Caring Sciences, Linköping University, Sweden.

[2] RISE Research Institutes of Sweden, Division of Bioeconomy and Health, Perception and Design Unit, Sweden.

[3] Department of Health Sciences, University of York, England

* Correspondence to: elizabeth.horlin@liu.se

Department of Health, Medicine and Caring Sciences, Division of Society and Health, Linköping University, 581 83 Linköping, registrator@liu.se, +46 13 28 10 00

CONTENT OF DIGITAL INTERVENTION TESTED IN THE PARENT TRIAL

The digital intervention aimed to target improving motivation and self-efficacy, as well as teaching new skills and addressing environmental constraints - which are understood to improve the likelihood of successful behaviour change, including for changing one's drinking. Although the evidence is not yet strong on how to best manipulate the aforementioned components with respect to alcohol consumption, promising actions to promote include those that focus on behaviour substitution, problem solving, goal setting, review of behavioural goals, self-monitoring, and normative feedback. Thus, modules included in the digital intervention revolved around these activities.

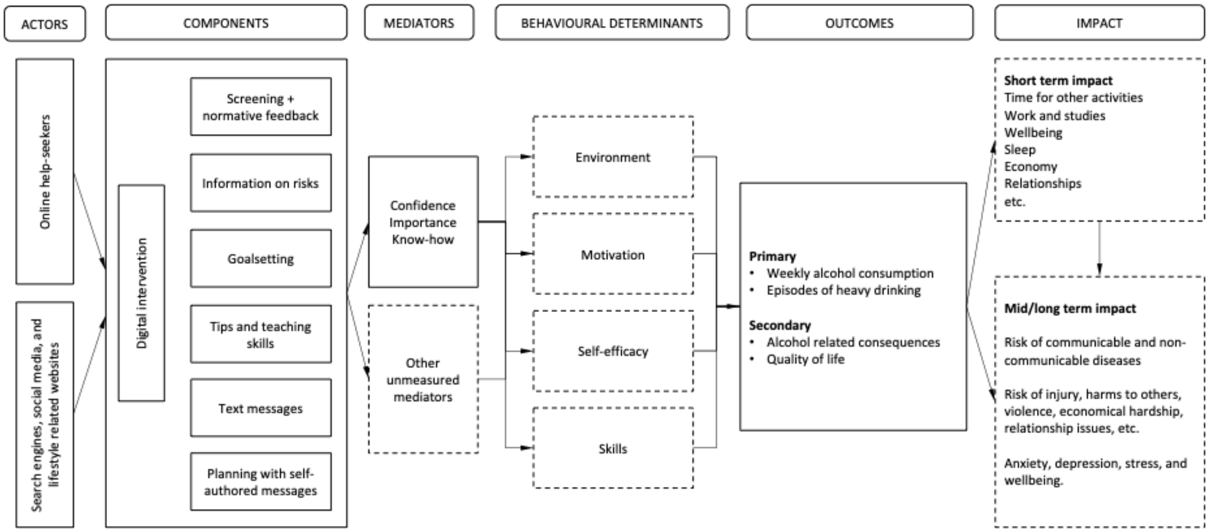

The core element of the digital intervention was a text message sent to participants each Sunday afternoon. The text message included a prompt to self-monitor one's current alcohol consumption, and a hyperlink to a web-based screening tool. Those who decided to click on the link were first screened with respect to total alcohol consumption in the past week, and frequency of episodes of heavy drinking the past month. Thereafter, they were given access to a personalised support tool which consisted of six modules contained in a web-based dashboard. The modules were:

1. Normative comparison of the participants' current consumption compared with others of the same age group and gender (based on data from Sweden), and risky drinking classification.

2. Information about some of the risks from drinking alcohol, including risk of disease, how it may affect children in proximity, driving, and other negative consequences. This module also allowed participants to simulate how different levels of consumption affected risk of cardiovascular disease.

3. One module allowed participants to create a plan which they could use when facing an environmental or behavioural trigger (e.g., going to the pub). This module asked participants to write a text message to themselves and pick a time and date for when they wanted to receive this message in the coming week (up to 3 times).

4. General tips to strengthen participants' know-how on how to reduce their consumption was given in one module. The tips included suggestions to create prompts or cues as reminders that they have committed to reduce their drinking, as well as suggesting that participants practice a new behaviour and substitute

their current behaviour with a different one (e.g., replacing at least two alcoholic beverages with non-alcoholic beverages each week). The tips also concerned identification of relapse triggers and barriers, avoiding social cues for drinking, and environmental restructuring (e.g., avoid keeping alcohol at home).

5. One module showed participants' consumption over time (with data from the weekly assessments). Participants could set a goal for their consumption, which would then show up graphically in the chart. This allowed participants to set and review their own goals while also visualising the discrepancy between their current consumption and their goals.

6. On Wednesdays, Fridays, and Saturdays, participants received additional text messages with content aimed to increase motivation and skills. Participants could also opt for additional text messages to be sent on the Mondays, Tuesdays, and Thursdays. The messages sent were a refinement of a previously developed set that was created through formative development and reported previously.

TABLE S1: CODEBOOK DEVELOPED FROM QUALITATIVE DATA IN PRESENT STUDY

Group ("Theme")	Description	Code	Explanation
Encouragement style	Ways that the individuals attempt to encourage or hype themselves into starting, or continuing, progress towards reduction, moderation, quitting etc.	*Pride/general self-belief*	Mentions being proud of their progress or that they have faith in their ability to achieve what they intend to
		Self-care	Mentions or implies they want to take better care of themselves more generally, or that they wish to go easy/be gentle towards themselves
		Self-control	Mentions or implies needing or wanting to exert self-control in order to stick to a plan, goal, or behaviour. Text that implies they are thinking in a willpower mindset. Includes statements such as "Think!" and implications that self-restraint is needed.
Level of awareness<br><br>(Awareness)	Messages that suggest some level of awareness or internal reflection on their past, present, or future situation and how these are related to or affected by their drinking.	*Specified consequences*	Mentions or implies understanding that their drinking has/will have negative consequences for specified future activities/goals/desires or lead them to behave in ways they wish to avoid (e.g., make a fool of myself on social media). Also relates to positive consequences stemming from

			changing their drinking (e.g., spending more time with my kids). Mentions other behaviours e.g., smoking, being related to or affected by drinking behaviour.
		Broad awareness	Mentions or implies recognition that they are dissatisfied with their current situation, or that they want their future situation to be different to how things are now but this is not explicitly connected to other behaviours or consequences of their drinking
Reminders or reasons/motivators to reduce/quit<br><br>(Reasons)	Person is providing or listing specific reasons they want to change their drinking behaviour, or seem to be reminding themselves of the reason they wanted to change	*Appearance*	Beauty or external appearance as a motivator
		Health – exercise	Motivation to exercise more; implication that drinking affects their ability to do so
		Health – general avoidance of negative physical/emotional wellbeing	Health concerns or goals in relation to drinking behaviour; wanting to avoid negative physical health outcomes or emotions rather than aiming for positive ones
		Health – general intention/desire to improve physical or emotional wellbeing	Health concerns or goals in relation to drinking behaviour; wanting or aiming for positive improvements in physical health or emotional wellbeing rather than avoiding negative ones
		Health - secondary prevention	Names a specific health issue, e.g., blood pressure, that is in some way affected by their drinking or serves as a specific reason to change their drinking behaviour
		Health – weight	Motivation to lose/not gain weight

	their drinking behaviour in the first place	*Mental health*	Specifies some mental health state that is affected by drinking; improved mental health as a motivator to reduce or stop drinking
		Money	Mentions financial concerns as a motivator to reduce alcohol, or money worries more broadly
		Other people	Mentions other people (not necessarily by name), could be groups of people; social reasons to reduce or other people as a motivator to reduce/stop
		Other people – children	Specifically mentions children as a reason to reduce drinking
Strategy to reducing/ moderating/ quitting<br><br>(Strategy/goal)	Use of goal setting and/or other methods, plans, or strategies for achieving those goals are mentioned or described.	*Non-specific goal*	Mentions or implies having a goal but does not define that goal (e.g., lose weight vs lose 10kgs, drink less vs drink 2 glasses) or is vague about what that goal is (e.g., want to have a good life); mentions or implies wanting to reach a vague or unspecified position (of their own desire/design) in the future (e.g., I want to have a bright future)
		Limit opportunity	Mentions avoiding settings or scenarios where opportunity for drinking is high, including not buying alcohol to have at home.
		Moderation strategy – days	Mentions drinking only on certain days as a way of reducing drinking, implies planning drinking only on certain days as a method of reduction

		Moderation strategy – units	Mentions drinking a certain number of drinks as a way of reducing drinking, setting a specific number of drinks as a (max) target, implies planning to count glasses as a strategy to reduce or moderate drinking
		Moderation strategy – time	Using time as a means to reduce consumption, such as not drinking until a certain time of day (e.g., don't drink until after dinner), drinking slower (e.g., drink it slowly) etc.
		Alternative to drinking	Mentions engaging in activities other than drinking, directly replacing alcohol with other activities, includes sleeping or going to bed early.
		Substitution with alcohol replacement	Mentions replacing alcohol with some kind of alternative, including water (e.g., every other drink is water)
Timescale	Mentions or implies employing change in either the shorter or longer term, either in the form of actions, intentions, or goals. Includes messages to not drink today, wait until tomorrow, wait a week, think about how one could feel in 20 years, and so on.	*Longer-term thinking*	Mentions reducing or abstaining for a longer period of time. Mentions or implies longer-term goals of moderation or abstinence, or has longer term goals that could/would be negatively affected if they don't change
		Shorter-term thinking	Mentions reducing or abstaining for a short period of time; implies taking an action or intending to do so to reduce drinking in the short term. Specifies a short-term goal that either necessitates reducing drinking or that their goal is to reduce in the short term specifically
